# Rhupus syndrome presenting with anterior scleritis and lupus nephritis: A case report

**DOI:** 10.1177/2050313X261467123

**Published:** 2026-07-11

**Authors:** Myrah Sheriff, Veena Patel

**Affiliations:** 1 University of Texas at Austin Dell Medical School, TX, USA; 2 Department of Internal Medicine, University of Texas at Austin Dell Medical School, TX, USA

**Keywords:** rheumatology, lupus, rheumatoid arthritis

## Abstract

Rhupus syndrome is a rare disease that presents with features of both rheumatoid arthritis and systemic lupus erythematosus. It is typically characterized by a rheumatoid arthritis pattern of symmetric, polyarticular small joint pain and swelling along with features of lupus such as photosensitive skin rashes and hematologic abnormalities. The disease is rare and inclusion criteria are mixed, but diagnosis is generally based on clinical features in conjunction with positive serologic lab results for both diseases. Severity can vary from benign to severe manifestations, rarely including anterior scleritis. We describe a case of a 20-year-old female with recently diagnosed inflammatory arthritis who presented with severe bilateral anterior scleritis and lupus nephritis, who achieved complete remission following treatment with high-dose intravenous glucocorticoids.

## Introduction

Rhupus syndrome is a rare condition characterized by the overlap of rheumatoid arthritis (RA) and systemic lupus erythematosus (SLE) features, affecting approximately 1.3% of hospitalized SLE patients. In most cases (84% of patients), the features of RA present first, followed by a milder spectrum of SLE involvement, as characterized by lower rates of renal and neurological involvement.^1^ Inflammatory arthritis is the main feature of rhupus, and usually presents similarly to RA with symmetric, polyarticular small joint pain and swelling, and can vary from mild, nondeforming arthritis, to erosive disease and disability. Compared with RA, there are no significant differences in the frequency of joint pain, morning stiffness, or joint tenderness.^2^ However, compared with SLE patients, erosive disease and nodules are significantly more common in rhupus patients. Compared to SLE patients, however, rhupus patients have less systemic and extra-articular involvement. The most common SLE features in rhupus patients include hematological abnormalities, skin and mucosal involvement, pericarditis, and cutaneous manifestations (e.g. malar rash, photosensitivity). Overall, rhupus patients demonstrate a clinical profile characterized by more severe RA-associated damage, milder SLE manifestations, and significantly less renal involvement (compared to SLE).

Affected individuals also have dual autoantibody profiles with elevated rheumatoid factor (RF), anti-cyclic citrullinated peptide antibody (anti-CCP), antinuclear antibody (ANA), and anti-double-stranded DNA titers (anti-dsDNA), sometimes accompanied by positive anti-Smith and low complement (C3/C4) levels.^2^ There is currently no specific literature documenting the association of rhupus syndrome and anterior scleritis, however, these ocular findings can be seen with RA. This could be due to the rarity of scleritis in association with SLE, and that rhupus patients tend to have milder SLE manifestations. A bilateral presentation of anterior scleritis is extremely rare in a patient with known SLE.

Anterior scleritis is a sight-threatening inflammatory disease of the sclera that predominantly affects middle-aged women. It is a predominantly unilateral condition, however, bilateral scleritis is significantly more associated with systemic autoimmune disease, including RA (15%–39% of cases), and very rarely SLE (<5%).^3^ Other autoimmune disease associations include granulomatosis with polyangiitis, inflammatory bowel disease, Bechet’s disease, Sjogren’s syndrome, and relapsing polychondritis.^3^ Differential diagnosis can also include rosacea, gout, and infectious causes, including herpes zoster, herpes simplex, syphilis, and Lyme disease.^3^

In the rare case of anterior scleritis as the initial symptom of SLE, it typically presents as unilateral, diffuse anterior scleritis, characterized by widespread scleral inflammation, injection, and severe, deep, radiating ocular pain that worsens at night.^4^ Diagnosis is primarily clinical and based on slit-lamp examination findings.

Lupus nephritis frequently presents as the initial manifestation of SLE, but it is exceedingly rare to present as the initial symptom in rhupus. It carries a mortality rate of 30% at 10 years after diagnosis.^5^ It is a glomerulonephritis characterized by proteinuria and red cell casts seen on urinalysis initially, but can progress to worsening kidney function and end-stage renal disease requiring dialysis. The aim of this manuscript is to report a rare presentation of rhupus syndrome with bilateral anterior scleritis and lupus nephritis, and to highlight the relevant diagnostic and management considerations.

## Case report

**Figure 1.**
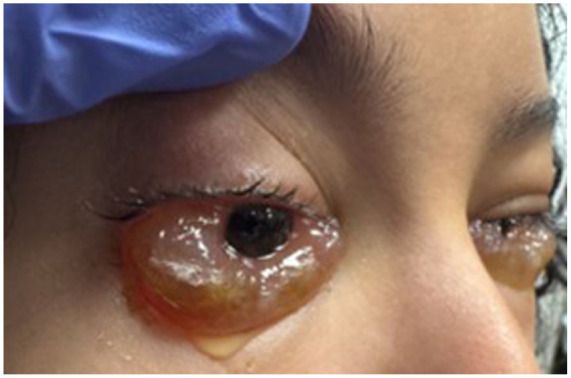
Initial findings of severe bilateral anterior scleritis before treatment.

A 20-year-old Hispanic woman initially presented with 4 months of symmetric joint pain in greater than 10 joints, including her hands, shoulders, elbows, knees, and toes, with associated swelling in her fingers and morning stiffness. She saw a rheumatologist and was found to have positive ANA 1:1280 nuclear, speckled pattern (immunofluorescence assay), dsDNA 1:40 (crithidia assay), anti-Smith (>8), anti-ribonucleoprotein antibodies (>8), and anti-cyclic citrullinated peptide (anti-CCP) 35 units (<20 normal), and was diagnosed with SLE. RF, Ro/La (SSA/SSB), erythrocyte sedimentation rate (ESR), and C-reactive protein (CRP) were all negative at the time of outpatient diagnosis. During this evaluation, she was also found to have a positive QuantiFERON gold, but had no symptoms concerning for active tuberculosis and a normal chest x-ray, thus she was treated for latent tuberculosis (LTBI). She was prescribed hydroxychloroquine and rifampin, but discontinued both after 1–2 weeks due to nausea. She was then started on 20 mg prednisone daily with improvement in her joint pain, but after tapering to 5 mg daily, she started to experience bilateral eye pain, redness, tearing, and blurry vision. Associated symptoms included hair shedding and chest discomfort. Her ocular symptoms rapidly worsened, and she was admitted to the hospital for a severe bilateral anterior scleritis flare (Figure 1). The patient had no significant past medical history, family history, or surgical history. She had no relevant risk factors for tuberculosis. On physical exam, the patient weighed 80 kg, and ocular exam revealed conjunctiva chemosis inferiorly, scleral injection with violaceous hue, and pain to palpation of the orbit superiorly.

Abnormal inpatient laboratory tests are as follows: urinalysis with large blood, three to five RBC/HPF (high power field), protein 100 mg/dL, occasional fine granular casts, 24 h urine protein: 1.8 g, C3: 28 mg/dL (normal: 74–148), C4: <2.9 mg/dL (normal: 14–39), dsDNA of 24 IU/mL (normal: 0–9), ESR 142 (normal: 0–20), and CRP 0.4 mg/dL (normal: <0.5 mg/dL). The patient had a normal complete blood count and creatinine. Due to proteinuria, the patient underwent a kidney biopsy, which revealed class III lupus nephritis, with full-house immunofluorescence staining, NIH activity index of 2/24.

Based on her initial presentation of pain and swelling in greater than 10 joints (including small joints) for over 4 months and positive anti-CCP, she meets the 2010 ACR/EULAR classification criteria for RA.^6^ In addition to the bilateral anterior scleritis with positive ANA and positive dsDNA, her diagnosis was updated to rhupus syndrome. The patient shared that finally having one diagnosis that explained her symptoms made a difficult experience feel more manageable. She was given 1000 mg IV methylprednisolone daily for 3 days without much improvement in ocular symptoms, so this was extended to 5 days in total. She also received prednisolone 0.1% ophthalmic drops four times daily and indomethacin 50 mg every 8 hours while hospitalized for refractory symptoms. Her creatinine remained normal and her kidney function was closely monitored. She was discharged on prednisone 60 mg daily and restarted on rifampin. Hydroxychloroquine was also started at a lower dose due to the previous nausea at 200 mg daily (⩽5 mg/kg/day), with plans to increase as tolerated to 400 mg daily. Due to her positive QuantiFERON gold, she was receiving treatment for latent TB, and therefore, steroid-sparing immunosuppression was not started at this time.

**Figure 2.**
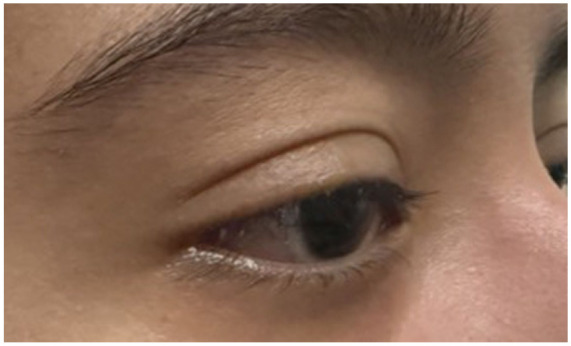
Right eye after receiving treatment with high-dose glucocorticoids in combination with indomethacin for 2 weeks.

She was seen for outpatient follow-up 2 weeks after discharge and her ocular symptoms had resolved (Figure 2). She was tolerating her medications well. After treatment with rifampin for 1 month, she was started on mycophenolate mofetil 1500 mg BID, continued on hydroxychloroquine, and placed on a prednisone taper.

## Discussion

Anterior scleritis is a rare symptom of RA (manifests in 2% of patients), but is exceedingly rare in SLE and rhupus syndrome, especially in the bilateral severe form.^7^ This case illustrates a favorable outcome in a patient with SLE who not only had a rare manifestation of the disease, but also a bilateral and severe presentation. Clinicians should maintain a high index of suspicion for rhupus rather than SLE in patients with such severe manifestations of anterior scleritis. Additionally, as this is a single-case observation, this report is limited by its retrospective design and low generalizability, and further controlled studies are preferred to confirm these findings.

Furthermore, despite this patient presenting with ocular symptoms, they also had class III lupus nephritis, demonstrated by proteinuria and confirmed by kidney biopsy. This case highlights the importance of multiorgan screening for those with a new diagnosis of SLE. Lupus nephritis can present as the initial manifestation of SLE and is more likely to develop within 5 years of SLE diagnosis.^5^ Severe ocular manifestations of SLE are also linked with lupus nephritis due to the shared common pathological processes of immune complex deposition and complement fixation. Those with newly diagnosed lupus, including individuals already demonstrating ocular findings, should undergo some screening for renal disease with urinalysis and spot urine protein/creatinine testing. If there is suspicion for lupus nephritis, a kidney biopsy is necessary to confirm the diagnosis.

This patient’s positive QuantiFERON gold and ophthalmologic findings made selecting the appropriate steroid-sparing therapy particularly unique. Initial treatment focused on treating the latent tuberculosis (LTBI) with rifampin while continuing an NSAID and oral corticosteroid until the patient could safely be started on steroid-sparing, induction immunosuppressive therapy at least 4 weeks after starting LTBI treatment.^8^ Additionally, mycophenolate mofetil 1500 mg BID was selected as an induction therapy for the lupus nephritis, which will also treat anterior scleritis (Supplemental Material).

## Conclusion

Rhupus, an overlap autoimmune disease of RA and SLE, very rarely presents with such a severe initial ocular presentation as seen in this case. However, if the differential diagnosis includes several conditions with overlapping clinical features, serologic testing may help distinguish between distinct disease states. Additionally, all patients with a new diagnosis of lupus or rhupus should be screened for lupus nephritis with a urinalysis with microscopy and random urine protein:creatinine ratio.

## Supplemental Material

sj-pdf-1-sco-10.1177_2050313X261467123 – Supplemental material for Rhupus syndrome presenting with anterior scleritis and lupus nephritis: A case report Supplemental material, sj-pdf-1-sco-10.1177_2050313X261467123 for Rhupus syndrome presenting with anterior scleritis and lupus nephritis: A case report by Myrah Sheriff and Veena Patel in SAGE Open Medical Case Reports
